# Supplementary material for: Naturally Occurring ZCCHC3 Variants Modulate Antiretroviral Activity in Cynomolgus Macaques
Source: Microbiol Immunol. 2026 Sep 4;70(11):543–53. doi: 10.1111/1348-0421.70088 (PMC13729731; doi:10.1111/1348-0421.70088)
Supplement: Supplementary file 1 [file MIM-70-543-s001.pdf]

Table S2. Frequency of amino acid substitutions identified in cynomolgus macaque ZCCHC3. Summary of amino acid substitutions identified in ZCCHC3 from 63 cynomolgus macaques. Eight independent ZCCHC3 cDNA clones from each animal were analyzed by Sanger sequencing. The table lists the amino acid substitutions identified, the number and percentage of animals carrying each substitution, and the corresponding animal IDs. Animals carrying the same substitution, either alone or in combination with other substitutions, were counted only once for that substitution. WT denotes animals whose ZCCHC3 amino acid sequence was identical to the Macaca fascicularis reference sequence (NCBI XP_005568656.1). Frequencies were calculated based on a total of 63 animals. An asterisk (*) indicates a premature stop codon (Q304 and A157*).

Amino acid substitution	No. of animals	Frequency (%)	Animal ID(s)
A120T	11	17.5	#10, 15, 26, 28, 35, 43, 44, 47, 54, 58, 61, 62
A118S	4	6.3	#11, 25, 47, 63
A31V	2	3.2	#18, 38
A79V	2	3.2	#22, 40
E306G	2	3.2	#24, 40
E8G	2	3.2	#38, 44
G69S	1	1.6	#10
S72G	1	1.6	#25
T73A	1	1.6	#37
G74W	1	1.6	#62
G76S	1	1.6	#9
A79T	1	1.6	#30
G95R	1	1.6	#9
K115R	1	1.6	#28
A131T	1	1.6	#9
E147K	1	1.6	#49
A157*	1	1.6	#61
E174G	1	1.6	#30
S191P	1	1.6	#29
S208G	1	1.6	#16
E210K	1	1.6	#22
Y228C	1	1.6	#10
Q234R	1	1.6	#55
G245W	1	1.6	#49
S247G	1	1.6	#38

V263M	1	1.6	#24
D264G	1	1.6	#59
D267G	1	1.6	#48
Q282P	1	1.6	#37
V285A	1	1.6	#42
G294W	1	1.6	#46
K297R	1	1.6	#62
Q304*	1	1.6	#27
G320A	1	1.6	#5
E332K	1	1.6	#33
G345D	1	1.6	#15
R351G	1	1.6	#26
R366Q	1	1.6	#20
L373P	1	1.6	#25
A393T	1	1.6	#61
L396P	1	1.6	#11
