# Supplementary material for: Naturally Occurring ZCCHC3 Variants Modulate Antiretroviral Activity in Cynomolgus Macaques
Source: Microbiol Immunol. 2026 Sep 4;70(11):543–53. doi: 10.1111/1348-0421.70088 (PMC13729731; doi:10.1111/1348-0421.70088)
Supplement: Supplementary file 2 [file MIM-70-543-s002.pdf]

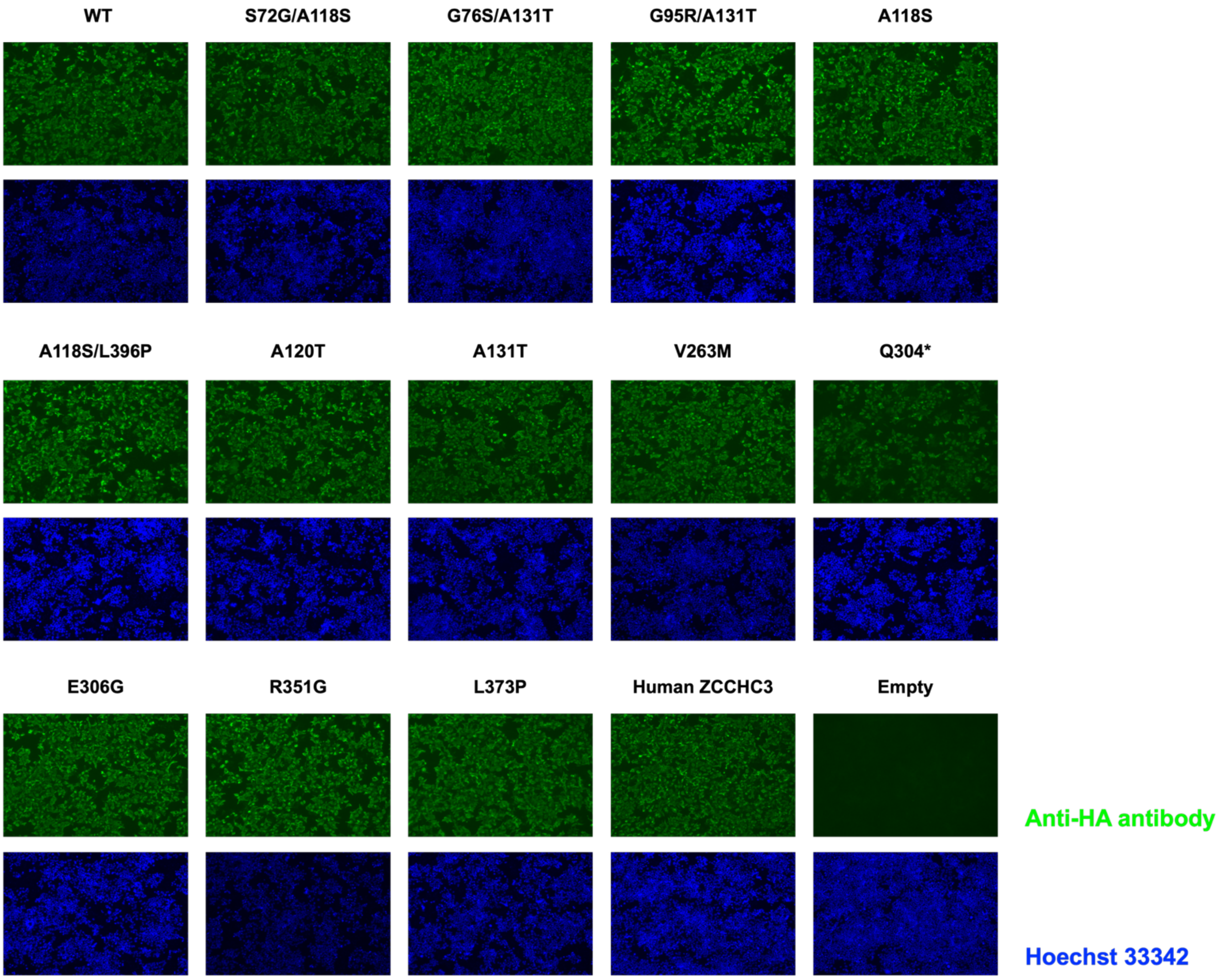

Figure S2. Subcellular localization of cynomolgus macaque ZCCHC3 variants.

Lenti-X 293T cells were transiently transfected with HA-tagged expression plasmids encoding wild-type (WT) or the indicated cynomolgus macaque ZCCHC3 variants, or human ZCCHC3. At 48 h post-transfection, cells were fixed and subjected to immunofluorescence staining using an anti-HA antibody (green). Nuclei were counterstained with Hoechst 33342 (blue). Empty vector-transfected cells served as a negative control. Representative fluorescence images are shown.
