# Supplementary material for: Naturally Occurring ZCCHC3 Variants Modulate Antiretroviral Activity in Cynomolgus Macaques
Source: Microbiol Immunol. 2026 Sep 4;70(11):543–53. doi: 10.1111/1348-0421.70088 (PMC13729731; doi:10.1111/1348-0421.70088)
Supplement: Supplementary file 3 [file MIM-70-543-s003.pdf]

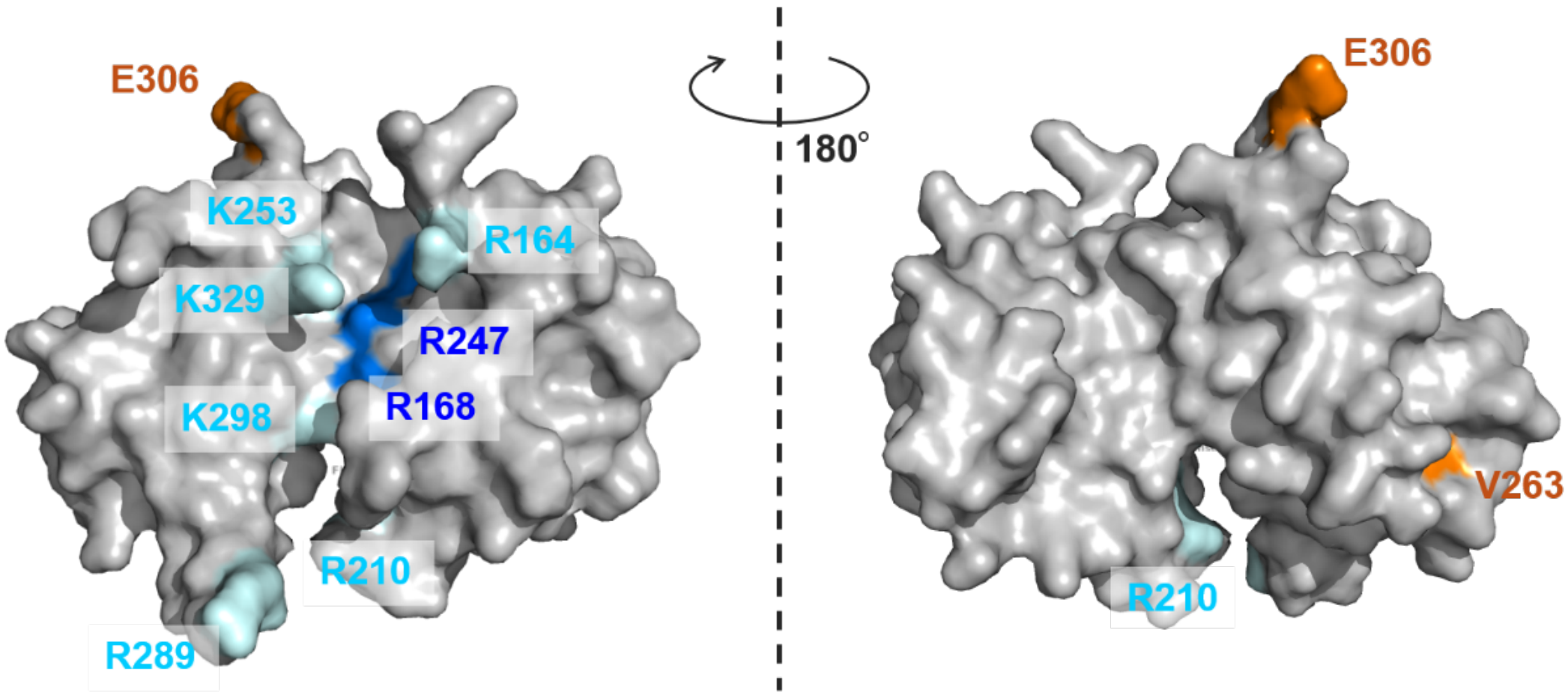

Figure S1. Locations of the ZCCHC3 amino acid substitutions on the three-dimensional structural model of the central fold domain.

The positions of the amino acid substitutions in the central fold domain (V263 and E306) are shown (orange). The basic residues forming the RNA-binding pocket (R164, R168, R210, R247, K253, K289, R298, K329 in human ZCCHC3) are labeled in cyan. Basic residues critically involved in the RNA-binding (R168 and R247 in human ZCCHC3 [16], corresponding to R167 and R246 in cynomolgus macaque ZCCHC3) are indicated in blue. V263 and E306 map to the face of the domain opposite the RNA-binding pocket.
