# Supplementary material for: Naturally Occurring ZCCHC3 Variants Modulate Antiretroviral Activity in Cynomolgus Macaques
Source: Microbiol Immunol. 2026 Sep 4;70(11):543–53. doi: 10.1111/1348-0421.70088 (PMC13729731; doi:10.1111/1348-0421.70088)
Supplement: Supplementary file 4 [file MIM-70-543-s004.pdf]

Table S3. Representative ZCCHC3 variants selected for functional characterization. Representative ZCCHC3 variants selected for functional analyses are listed together with their corresponding predicted structural regions. Variants were selected to represent the diversity of naturally occurring ZCCHC3 sequence variation identified in 63 cynomolgus macaques, taking into account their frequency, distribution across the predicted structural regions of the protein, and inclusion of single-substitution, double-substitution, and truncation variants. The frequency of each amino acid substitution is summarized in Table S2.

**Representative variant**	**Predicted protein region**
S72G/A118S	N-terminal disordered region
G76S/A131T	N-terminal disordered region
G95R/A131T	N-terminal disordered region
A118S	N-terminal disordered region
A118S/L396P	N-terminal disordered region / C-terminal region containing the zinc-finger motifs
A120T	N-terminal disordered region
A131T	N-terminal disordered region
V263M	Central fold domain
Q304*	Central fold domain
E306G	Central fold domain
R351G	C-terminal region containing the zinc-finger motifs
L373P	Zinc-finger region
