# Supplementary material for: Naturally Occurring ZCCHC3 Variants Modulate Antiretroviral Activity in Cynomolgus Macaques
Source: Microbiol Immunol. 2026 Sep 4;70(11):543–53. doi: 10.1111/1348-0421.70088 (PMC13729731; doi:10.1111/1348-0421.70088)
Supplement: Supplementary file 5 [file MIM-70-543-s005.pdf]

Table S1. Predicted localization of cynomolgus macaque ZCCHC3 variants.

Subcellular localization of wild-type (WT) and variant cynomolgus macaque ZCCHC3 proteins predicted using DeepLoc 2 based on their amino acid sequences. The predicted primary subcellular localization, together with the corresponding probabilities for nuclear and cytoplasmic localization, is shown for each variant. Human ZCCHC3 was included for comparison. All predictions were performed using the default parameters of DeepLoc 2.

Variant	Predicted localization	Nuclear probability	Cytoplasmic probability
WT	Nucleus	0.6258	0.3554
S72G/A118S	Nucleus	0.5765	0.4119
G76S/A131T	Nucleus	0.6753	0.297
G95R/A131T	Nucleus	0.6314	0.3423
A118S	Nucleus	0.6384	0.3397
A118S/L396P	Nucleus	0.6952	0.2865
A120T	Nucleus	0.7149	0.2705
A131T	Nucleus	0.6798	0.2988
V263M	Nucleus	0.6062	0.3725
Q304*	Nucleus	0.5091	0.4773
E306G	Nucleus	0.5592	0.4171
R351G	Nucleus	0.64	0.3439
L373P	Nucleus	0.6619	0.3111
Human ZCCHC3	Nucleus	0.5186	0.4447
